# Effect of cat litters on feline coronavirus infection of cell culture and cats

**DOI:** 10.1177/1098612X19848167

**Published:** 2020-04

**Authors:** Diane Addie, Lene Houe, Kirsty Maitland, Giuseppe Passantino, Nicola Decaro

**Affiliations:** 1 Institute of Comparative Medicine, University of Glasgow, Glasgow, UK; 2 Reg Acc, Felismir Katteklinik, Hvalso, Denmark; 3 Department of Veterinary Medicine, University of Bari, Bari, Italy

**Keywords:** Feline coronavirus, feline infectious peritonitis, FIP, FCoV infection, cat litter, virus inhibition, reducing virus load, Fuller’s earth, Bentonite, Dr Elsey

## Abstract

**Objectives:**

Feline infectious peritonitis (FIP) is caused by infection with feline coronavirus (FCoV). FCoV is incredibly contagious and transmission is via the faecal–oral route. FCoV infection, and therefore FIP, is most common in breeder and rescue catteries, where many cats are kept indoors, using litter trays. Whether it is possible to break the cycle of FCoV infection and reinfection using cat litters has never been investigated. The aim of the study was to examine the effect of cat litters on FCoV infectivity and virus load in multi-cat households, and transmission frequency.

**Methods:**

Fifteen cat litters were mixed and incubated with FCoV, centrifuged and the supernatants tested in vitro for the ability to prevent virus infection of cell culture. To test applicability of in vitro results to real life, virus load was measured in two households in a double crossover study of four Fuller’s earth-based cat litters by testing rectal swabs using FCoV reverse transcriptase quantitative PCR.

**Results:**

Four litters abrogated FCoV infection of cell culture, nine reduced it to a greater or lesser extent and two had no effect. One brand had different virus inhibitory properties depending on where it was manufactured. Fuller’s earth-based litters performed best, presumably by adsorbing virus. In the field study, there appeared to be less virus shedding on one Fuller’s earth-based cat litter.

**Conclusions and relevance:**

The in vitro study successfully identified cat litters that inactivate FCoV; such litters exist so do not need to be developed. Fuller’s earth-based litters best prevented infection of cell culture, but did not completely abrogate FCoV transmission in two multi-cat households. A dust-free clumping Fuller’s earth litter appeared to fare best, but virus shedding also varied on the control litters, complicating interpretation. Sawdust-based cat litters are not useful in FCoV-endemic households because they track badly and have a poor effect on virus infection.

## Introduction

Feline coronavirus (FCoV) is a highly contagious, enveloped, positive-strand RNA virus that is related to canine coronavirus (CCoV) and transmissible gastroenteritis virus of swine, forming with these viruses a unique species, *Alphacoronavirus-1*, within the genus *Alphacor-onavirus* (family Coronaviridae, order Nidovirales).^1^ Coronaviruses infect most species and are prone to high rates of recombination. Within Alphacoronavirus-1 species, FCoV exists in two different genotypes, FCoV type I (FCoV-I) and type II (FCoV-II), with the latter arising from recombination events between FCoV and CCoV.^2,3^

A minority of FCoV-infected cats go on to develop an immune-mediated inflammatory vasculitis known as feline infectious peritonitis (FIP), which is usually fatal. The majority of infected cats sheds virus in faeces for some months, then spontaneously cease shedding.^4^ However, recovered cats are then susceptible to reinfection.^5–7^ with the same, or another, strain of FCoV.^5^ Persistent infection with coronaviruses has been postulated to select for expansion of changes in tissue tropism and/or emergence of hypervirulent strains,^8^ and there is evidence that the emergence of FIP in a household is more likely where there is a higher virus load.^9^

FCoV has three major strategies for not going extinct: firstly, the majority of viral transmission is from transient virus shedders getting infected repeatedly;^4–7^ secondly, around 13% of infected cats become persistently infected healthy carrier cats,^4^ shedding a restricted strain of virus that the immune system of the cat presumably does not recognise;^5^ thirdly, the virus is moderately robust, being able to survive for up to 7 weeks when protected by faecal matter,^10^ thus indirect transmission is the major route of infection. The major source of FCoV infection is faeces,^4^ and most virus transmission is through sharing litter trays with an FCoV-infected cat. Prevention of FCoV infection (and therefore FIP) involves physical separation of infected and uninfected cats,^11^ although this is rendered more difficult by the highly infectious nature of the virus, and possible indirect transmission on fomites.

Whether it is possible to break the cycle of FCoV transmission by using cat litters that make it more difficult for FCoV to remain infectious has never been investigated. The first step in such an investigation was to examine the anti-coronaviral activity of currently available cat litters: the results of an in vitro study of these are presented in this paper. The second step was to test the effect of cat litters in the field; the recent advent of quantitative PCR (qPCR) has enabled measurement of virus load in rectal swabs and faeces,^12^ allowing assessment of the effect of cat litter, if any, on virus load and transmission. This is the first report of the effect of cat litter on coronavirus load in multi-cat households.

## Materials and methods

### Cat litters

Fifteen cat litters, representing each of the main commonly used formulations – Fuller’s earth; wood/sawdust pellets; silica gel; recycled paper and clay; compressed wheat grass; whole-kernel corn; and one chicken feed (Nutrena Nature), which is sometimes used as a cat litter in the USA – were tested in vitro (Table 1) and an additional four litters were tested in vivo.

**Table 1:** Effect of cat litters on ability of feline coronavirus (FCoV) to infect cell culture

Cat litter	Litter type	Cytotoxicity	FCoV titre/ ml
Virus control (no litter)	NA	NA	10^6^
Ever Clean Less Track (Clorox International)	Fuller’s earth	No	0
Tesco Value (Tesco)	Fuller’s earth	No	0
LitterPurrfect (imported by Costco UK)	Fuller’s earth	No	0
Cat Country USA (Mountain Meadows Pet Products)	Compressed wheat grass	No	0
Sophisticat (Steetley)	Fuller’s earth	No	10
Wood-based cat litter (Pets at Home)	Wood pellets	No	10^2^
Catsan (Masterfoods)	Softwood granules	No	10^2^
Snowflake (Ashton-under-Lyne)	Wood pellets	No	10^2^
World’s Best Original (Grain Processing Company)	Whole-kernel corn	No	10^3–4^
Tesco Premium (Tesco)	Recycled paper and clay	No	10^4^
Nutrena Nature (USA)	Chicken feed	No	10^4^
World’s Best Extra (Grain Processing Company)	Whole-kernel corn	No	10^4–5^
Litter Pearls (Crystal Clear Pet Products)	Silica gel	No	10^4–5^
Cat Country UK (Mountain Meadows Pet Products)	Compressed wheat grass	No	10^6^
Cat Country Europe (Mountain Meadows Pet Products)	Compressed wheat grass	No	10^6^

The reduction of FCoV infectivity for cell culture in the supernatant of various cat litters is shown, in decreasing effect against the virus. The titre of the virus inoculum was 10^6^/ml: four cat litters caused the virus titre to reduce to 0 (ie, prevented infection of the cell culture), eight litters and the chicken feed reduced the FCoV titre to varying extents, and two litters had no effect on virus titre. None of the products tested were toxic to the cell culture

NA = not applicable

The real-world study used the households customary cat litters as control litters, and these both happened to be Fuller’s earth based: in household H it was Catrine (Kruuse), labelled litter A; and in household L it was (Ever Clean; Clorox International), labelled litter B. The trial litters were Dr Elsey Cat Attract (labelled litter X) and Dr Elsey’s Ultra, USA (labelled litter Y): these two litters were also Fuller’s earth based and litter X included herbs to attract cats to use it, and is non-tracking.

### Assay of FCoV inhibition by cat litter

The Wellcome strain of FCoV,^13^ at a titre of 1.5 × 10^6^/ml was used and 3 ml put onto 1 g of each cat litter in a tube. The tubes were rotated at room temperature for 2 h. After spinning at 8316 *g* for 10 mins, the supernatant was spun in a microfuge at 18,078 *g* for 5 mins to remove any remaining cat litter. The supernatant was filtered through a 0.45 µm filter (Pall Life Sciences) and 10-fold dilutions were made. Two-hundred µl of each dilution was applied to a well of feline embryo A cells^14^ plated the previous day at 2 × 10^5^ cells per well of a 12 well plate and cultured overnight at 37°C. Inoculae were left in contact for 4 h then removed and replaced with Dulbecco’s cell culture medium (Gibco Thermo Fisher Scientific) with 10% fetal bovine serum (PAA Laboratories). The cells were then placed at 37ºC and examined after 48 h for evidence of cytopathic effect. Supernatant from each cat litter was titrated in 10-fold dilutions in triplicate. Three millilitres of medium without FCoV were put onto 1 g of cat litter and treated as described above to control for a possible cytotoxic effect of the cat litter. Virus titres were assessed by the 50% end-point calculated according to the method of Ramakrishnan.^15^

### Cats

#### Household H

Household H was a Maine Coon breeding household, which had experienced FIP deaths among the kittens and cats. Cats were kept mainly indoors, although there was a small outdoor run available in the summer. The cats normally mixed together, but queens were isolated in a room of the house during kittening.

The cat litter used from January 2009 in this household was Fuller’s earth based: Catrine (Kruuse); therefore, this was the control litter for that household, and was labelled litter A. Rectal swabs were taken over a 3 year period from up to 41 cats or kittens by one of the authors (LH) for FCoV reverse transcriptase-qPCR (RT-qPCR) testing (see Table H in the supplementary material).

#### Household L

Eight adult cats (L1–L7 and L9) in one household were naturally infected with FCoV (see Table L in the supplementary material). During the period of the study two kittens (L8 and L10) were introduced, and cat L9 died. The household comprised five Maine Coon, three Somalis, one Norwegian Forest Cat and one domestic (sometimes called European) shorthair cat. All cats were kept indoors, although they had access to an outside run. The cats lived together, mixed freely and shared litter trays.

All of the litter trays in the household contained a Fuller’s earth-based cat litter (Ever Clean; Clorox International): this litter was deemed to be the control litter of that household and is referred to as cat litter B henceforth. Rectal swabs were taken by one of the authors (LH) on 14 occasions over a 3 year period for FCoV RT-qPCR testing.

### FCoV RT-qPCR

Specimens were homogenised (10% w/v) in Dulbecco’s modified Eagle’s medium and subsequently clarified by centrifuging at 2500 *g* for 10 mins. One hundred and forty microlitres of the supernatants were used for RNA extraction by the QIAamp Viral RNA Mini Kit (Qiagen), following the manufacturer’s protocol, and the RNA templates were stored at –70°C until their use.

FCoV RT-qPCR was performed as previously described,^12^ with minor modifications.^16^ The RT-qPCR target gene was the 3′ untranslated terminal region (UTR), which is conserved among FCoVs. In brief, a one-step method was adopted using Platinum Quantitative PCR SuperMix-UDG (Invitrogen) and the following 50 µl mixture: 25 µl master mix, 300 nM primers FCoV1128f (GATTTGATTTGGCAATGCTAGATTT) and FCoV1229r (AACAATCACTAGATCCAGACGTTAGCT), 200 nM of probe FCoV1200p (FAM- TCCATTGTTGGCTCGTCATAGCGGA-BHQ1) and 10 µl template RNA. For absolute quantification, in vitro synthesised RNA transcripts of the 3′ UTR were used. Duplicates of log_10_ dilutions of standard RNA, representing 10^1^–10^9^ copies of RNA/µl of template, were analysed simultaneously in order to obtain a standard curve. The thermal profile consisted of incubation with UDG at 50°C for 2 mins and activation of Platinum Taq DNA polymerase at 95°C for 2 mins, followed by 45 cycles of denaturation at 95°C for 15 s, annealing at 48°C for 30 s and extension at 60°C for 30 s. Samples in which no amplicon was detected by cycle 45 were deemed to be negative.

### Field study design

Households with existing endemic naturally acquired FCoV infection in their cats were identified. The field study was blinded as far as possible: the scientists performing the FCoV RT-PCR were blinded to the purpose of the study. A field study is fraught with many interactive variables, such as the number of cats in the household and the frequency of de-clumping litter trays. Therefore, a crossover design was used, using the households and individual cats as their own controls, so that variables such as age of the cat, number of cats in the household, concurrent stress, and so on, would be as controlled for as practically possible.

Virus shedding was monitored within the households for a baseline period of 9 months (to identify possible carrier or resistant cats), then trial cat litters were used for 2 month periods each, with resting periods on the control cat litters between trials. Data from persistently infected cats (as defined by Addie and Jarrett^4^) were to be excluded because carrier cats shed virus, regardless of the cat litter being used. However, data from cats housed with such cats would be useful. The rare occurrence of FCoV-resistant cats was also taken into account,^4^ as they would not shed virus regardless of the cat litter and the presence of these cats would have also skewed results.

### Statistical analysis

Fisher’s exact test (two-tailed) was carried out using the statistics package in Excel (Microsoft Office 2007). The cat litters that the household normally used (A and B) were used as control litters to generate an expected range of virus shedding, against which cat litters X and Y were compared. Aside from the cat litter being used, there are many variables that can affect virus load in a household, for example the presence of kittens, how often litter boxes are cleaned, frequency of vacuuming, etc; the households acted as their own controls in a crossover study. In the virus transmission analysis, all non-carrier, non-resistant cats that had a negative rectal swab followed by a subsequent test were able to be included; if, in the unlikely event that intermittent virus shedding occurred, the effect would be neutralised by being equally as likely on the control litter as on the trial litter.

The null hypothesis of no difference between cat litters was rejected at the 5% significance level if the test’s *P* value was <0.05.

## Results

### In vitro effect of cat litter against FCoV

The in vitro effects of cat litter against FCoV are presented in Table 1. No cat litter caused cytotoxicity. Three commercially available Fuller’s earth-based cat litters totally abrogated viral ability to infect cell culture, and one considerably reduced the virus titre. One wheat grass litter prevented cell culture infection. Seven cat litters and the chicken feed reduced the FCoV virus titre, and two cat litters had no effect on FCoV.

A pelleted western red winter wheat grass product (Cat Country; Mountain Meadows Pet Products) had different virus inhibitory activities, depending on whether it was manufactured in the USA (where it had good virus inhibitory activity) or Europe or the UK (where it had no ability to inactivate FCoV).

#### Household H

**Figure 1:**
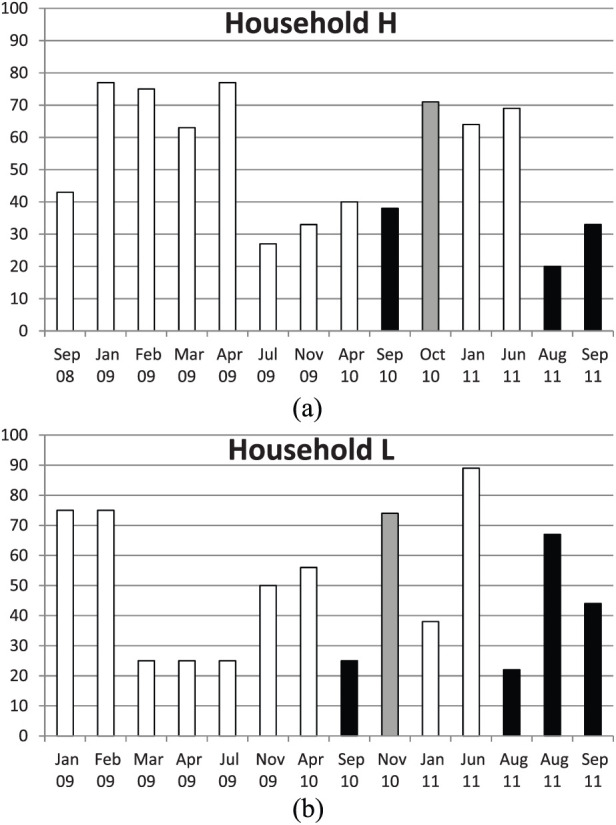
(a) Percentage of cats shedding feline coronavirus (FCoV) on crossover trial of cat litters A, X and Y in household H. (b) Percentage of FCoV-positive samples on crossover trial of cat litters B, X and Y in household L. Cats in households H and L were sampled on various occasions while using control cat litters A and B (white bars) and test litters X and Y (black and grey bars, respectively). This figure shows that the percentage of cats shedding FCoV was lower on cat litter X when compared with litters A, B or Y in a crossover study involving two households, H and L. It can be seen that the percentage of cats shedding FCoV decreased in both households when cat litter X was introduced, increased when the cat litter was changed from X to Y, then decreased again on litter X in both households. The difference between cat litters X and A was statistically significant (*P* <0.05), but the difference between X and B was not (*P* >0.05). Cat litter Y was not significantly different from litters A or B (*P* >0.05). All of the cat litters were Fuller’s earth based, but cat litter X was formulated to be dust-free and so to track less. The possibility that the reduction in virus shedding was related to summertime, as opposed to the cat litter, could not be excluded

As shown in Table H in the supplementary material, 41 cats and kittens were tested at various times, but, unfortunately, owing to the high turnover of cats and kittens, large numbers of sequential samples were only generated for 13 cats. During the study, cats were euthanased because of severe gingivostomatitis, to which Maine Coon cats are susceptible. Cat H14 was euthanased owing to suspected FIP. Results of FCoV RT-qPCR testing on rectal swabs is given in Table H in the supplementary material and Figure 1(a). No FCoV carrier cats were identified in this household, but Cat H17 was identified as being a possible FCoV-resistant cat.

#### Household L

The virus shedding results of the 10 cats in household L are shown in Table L in the supplementary material and Figure 1(b). There were no FCoV carrier cats in this household. Cat L5 appeared to possibly be a resistant cat, but then began shedding small amounts of virus. There were insufficient data for cat L9 which died; therefore, these results were omitted from analyses involving litter X.

### In vivo effect of four cat litters on the percentage of cats shedding FCoV at each sampling

A prospective crossover blinded controlled study was performed in the cats in households H and L, where two Fuller’s earth-based cat litters were habitually used: Catrine (Kruuse) in household H and Everclean (The Clorox Company) in household L. These control cat litters were labelled A and B, respectively. The cats were monitored for coronavirus shedding on seven occasions, then anonymised test litters were introduced: Dr Elsey Cat Attract (labelled litter X) and Dr Elsey’s Ultra, USA (labelled litter Y). A short duration pilot crossover was initially performed to establish whether X or Y was of greater interest; when X appeared to have an effect on the frequency of FCoV shedding, but litter Y did not, a longer period on litter X was undertaken after a resting period on the control litter (A or B) of each household. The results are shown in Figure 1 and full details are available in Tables H and L in the supplementary material.

The percentage of cats shedding FCoV appeared to decrease in both households on both occasions that cat litter X was used (Figure 1); however, the decreases were only statistically different in household H (against control litter A) and were not statistically significant in household L (against control litter B). Sample size in household L was insufficient to claim any differences between individual sampling times (all *P* >0.05). When all results for samples from household H cats H1–13 (ie, cats with sufficient samples to act as their own controls) were pooled, there were 54 (52%) positive samples and 49 (48%) negative samples on the control litter (A). On cat litter X there were five (26%) positive samples and 14 (74%) negative samples (*P* = 0.046). In household H, and counting all cats tested at both samplings, the changeover from litter A to X from April to September 2010 was not significantly different (*P* >0.05), but in the summer of 2011, when results from cats H30–41 were included with H1–4 samples, and were analysed solely for the switchover from litter A to litter X, there were 11 positive and five negative samples on litter A and eight positive and 22 negative on litter X, (*P* = 0.011).

The percentage of rectal swab samples that were positive and negative in household L is shown in Figure 1(b). In total of all sampling times, there were 33 (50%) positive samples and 33 (50%) negative samples on the control litter (B). On cat litter X there were 12 (38%) positive samples and 20 (62%) negative samples. The difference between X and B was not statistically significant (*P* >0.05).

#### Cat litter Y

In household H, using cat litter Y, there were 10 (71%) positive and four (29%) negative samples. Cat litter Y was not statistically different from cat litter A (*P* = 0.26). In household L, using cat litter Y there were six (75%) positive and two (25%) negative samples. Cat litter Y was not statistically different from cat litter B (*P* = 0.15). Pooling results from both households, cat litter X was significantly better than cat litter Y (*P* = 0.0148). However, in the previous year (2009) in both households, and in 2008 in household H, there was a significant increase in virus shedding in the first winter sampling compared with the summer samples (Figure 1a).

#### Infection/reinfection events were fewer on litter X vs the control litters

For this analysis, all data from the two households were used to find out if there were fewer new virus shedding episodes on cat litter X vs the two control cat litters, A and B (ie, results from cats that had been excluded from the crossover study were able to be included in this analysis.) Cat litters A and B could not be compared with each other because they were used in different households.

Positive samples that occurred after a negative sample (virus titre 0 in the supplementary tables) were counted, assuming that a new infection or reinfection of the cat had occurred (as the duration of immunity to FCoV reinfection is short-lived).

In household H, cats on cat litter A remained uninfected on 29 (50%) occasions and began shedding virus on 29 (50%) occasions; on cat litter X cats remained uninfected 18 (75%) times and became infected six (25%) times (*P* = 0.039). Results of cat H17 were not included because it was possibly a resistant cat.

In household L, cats on the control litter B stayed uninfected on 16 (48%) occasions and began shedding virus on 17 (52%) occasions; on cat litter X cats remained uninfected eight (57%) times and on six (43%) occasions cats began shedding FCoV after being negative (*P* = 0.75).

In summary, there were fewer reinfection events on cat litter X than on litters A or B, but not statistically significantly so on litter B.

## Discussion

FIP is one of the major infectious causes of feline death,^17^ and especially affects cats from multi-cat environments, such as cat breeders, where cats and kittens are exposed to high virus loads. At the time of writing, there is only one commercially available vaccine against FIP (Felocell-FIP; Pfizer/Zoetis),^18–21^ and it is used when kittens are 16 weeks old. However, maternally derived antibodies to FCoV wane when kittens are 4–7 weeks of age,^22^ leaving kittens susceptible to infection before vaccination can be performed. Kittens need to be protected from FCoV infection from the time of weaning until they are old enough to be vaccinated or rehomed; we decided to approach the problem at the source of infection – cat faeces. Our hypothesis was that if FCoV could be destroyed at source – either by a drug, within the cat’s intestines where the virus replicates, or immediately upon evacuation from the cat – then transmission of virus, and therefore FIP, would be prevented. The possibility of anti-coronavirus drugs is very real and their use in cats has recently been reported,^23–25^ but it will be some time before such drugs become approved for veterinary use.

We turned our attention to the possibility of developing an anti-coronavirus cat litter, and were surprised to find that some commercially available cat litters already had the ability to inhibit FCoV infection in vitro; ie, they prevented infection of cell culture. Three of the four litters that had the most effect were based on Fuller’s earth, raising the possibility that Fuller’s earth itself may have virus inhibitory properties. As Fuller’s earth is an inert substance that binds proteins and fats, it is possible that those Fuller’s earth-based cat litters prevented cell culture infection by binding the virus rather than actually killing it (Marian Horzinek, personal communication).

In vitro testing was useful to identify cat litters of interest, and for ruling out cat litters with no virus inhibitory properties (eg, those based on sawdust) but did not emulate real life, where the virus is protected by faecal matter. Virus-containing cat faeces could not be used in the experiments because of the difficulty of growing most wild strains of FCoV in cell culture and because, in addition to contamination difficulties, it would not have been feasible to obtain enough faeces to standardise the viral quantity; therefore, field experiments were undertaken.

The plan of the prospective controlled crossover field studies was to identify suitable households where FCoV was endemic, then determine whether intervention with a trial cat litter made any difference to virus transmission and virus load in the household. Four households volunteered for the study, but two had to be excluded owing to lack of FCoV transmission in the pre-trial study period (data not shown).

Two households yielded results for four different Fuller’s earth-based cat litters: litters A, B, X and Y. Cat litter X appeared to reduce virus shedding in both households on four occasions when it was used. However, occasions of use of litter X all fell around August and September, begging the question of whether it was the cat litter or the time of year that caused the reduction in virus excretion. One possible explanation might have been the presence of kittens in household H at certain times of the year: FCoV shedding tends to be higher in kittens and young cats than in older cats.^26^ However, the phenomenon was also seen in household L, which was not a breeding household. Another explanation could have been the use of small outdoor runs during the summer months and that possibility could not be excluded; the percentage of cats shedding virus in both households on the control litters in July of 2009 was low, although in household L the reduction in cats shedding FCoV occurred as early as March, which is certainly not summertime in Denmark. The possibility that the reduction in virus shedding was related to summertime, as opposed to the cat litter, might be coincidental, but cannot be excluded. Future cat litter studies will need to include the possibility of seasonal variations in virus shedding. A third possibility was that new FCoV quasi-species were appearing sporadically, causing an increase in virus shedding, which then decreased as cats acquired immunity to the new virus, the timing being coincidental to the cat litter changes.

Cat litter X has very little virus tracking compared with the other cat litters. For the purpose of demonstrating the in vivo effect of cat litters on FCoV infection and transmission, the ideal control cat litter would have been a sawdust-based litter, with little anti-FCoV activity and high tracking: it is very likely that a change to litter X would have had more effect if the control cat litters had been sawdust based, but given the possible lethal consequences of FCoV infection, it would not have been ethical to advise the households to change to such a cat litter when they were already using a Fuller’s earth-based litter.

Although Fuller’s earth-based cat litters prevented cell culture infection in the laboratory, they failed to totally prevent virus transmission in a real-life situation. There are possible explanations other than reinfected to explain these results; for example, it is possible that the appearance of reinfection was an artefact of our assay – perhaps failing to detect very low levels of virus shedding, and while this cannot be ruled out, it is not thought to be likely. Intermittent virus shedding by cats has been reported previously,^4^ but that study used an earlier version of RT-PCR technology than used in the present study and we have not seen intermittent virus shedding using newer technology. Another possible explanation is that rather than being reinfected, the cats were showing recrudescence of latent virus; however, a previous study in which cats were naturally stressed (by pregnancy) or given methylprednisolone acetate to immunosuppress them failed to demonstrate re-shedding and concluded that latent or sub-detectable infections likely do not occur.^7^ Another consideration was that a negative result did not necessarily mean that virus infection was inhibited by the litter, as an immunity – although shortlived – remains after the shedding period.

The main purpose of this study was to identify commercially available cat litters with inhibitory effect on FCoV, and to discover which, if any, could be used to prevent FCoV transmission; the pilot study succeeded in identifying cat litters of interest on which to base a controlled field trial. A field trial was essential because in the real world the virus is protected by faeces and is presumably not as easy to inactivate as in a liquid phase in vitro.

There are many factors that could affect whether or not a virus-binding cat litter will help to contain FCoV infection in the field. Firstly, the amount of virus shed by some cats is considerable:^7,27^ cats in a rescue shelter in the USA were found to shed billions of virus particles per gram of faeces and virus shedding increased millions-fold in some cats after a week in the shelter.^27^ Where millions of viral particles are being shed, even if 99% of the virus was being inactivated by a cat litter, then hundreds of thousands of infectious viral particles will still remain to infect other cats. Secondly, some litters track more than others and our study indicated that tracking was an important quality; cat litter X, which is claimed to be 99% dust-free, performed consistently better than the other three Fuller’s earth-based litters examined. Thirdly, the knowledge that a litter can inhibit virus transmission may alter human behaviour, perhaps reducing de-clumping frequency. Fourthly, not all cats use a litter tray or fully cover their faeces.

Although cat litter X did appear to reduce virus load, it did not totally abrogate virus transmission; therefore, it is likely that in order to completely prevent virus transmission in a household of cats, it is necessary to not only have a virus inhibitory and non-tracking cat litter, but to also prevent sharing of litter trays (eg, by allocating litter trays to individual cats and preventing their use by other cats in the household, which could be done by placing the trays behind doors with microchip-operated cat flaps). It is important that people with multi-cat households do not assume that a cat litter can totally prevent FCoV transmission; in one of the two households whose data were not shown, after we ceased monitoring (because the cats were all FCoV negative) a purebred kitten was introduced and one of the resident cats developed FIP and died. The household was using World’s Best Original Cat Litter, which has some virus inhibitory properties and minimal tracking, but that was not enough to prevent FCoV transmission from the introduced kitten.

## Conclusions

We identified litters with good in vitro activity against FCoV; therefore, there was no need to develop an anti-coronavirus cat litter. This small study was most informative for the design of a future large field trial; we successfully identified cat litters that were most likely to succeed or fail. Fuller’s earth-based litters fared best in vitro, and seemed to reduce, but did not abrogate, FCoV transmission in field studies.

Three of the four best-performing litters in our in vitro trial were based on Fuller’s earth, but FCoV is highly contagious and Fuller’s earth is a dusty litter that tracks on human and feline feet. A Fuller’s earth-based litter that tracked minimally appeared to decrease virus load in households better than did similar cat litters; therefore, the ability of a litter not to track was an important factor in FCoV control in multi-cat households. The crossover study design reduced the effect of variability in the response of the individual cat to FCoV, enabling the cat itself to act as its own control, and controlled for myriad environmental variables, such as owner behaviour and endemic FCoV type.

Until further information becomes available, best advice for FCoV prevention is that, where possible, cats be allowed access to outdoors to defaecate, or that separate litter trays are used for each cat.

## Supplemental Material

Cat_litter_Supplementary_Material_Apr_2019 – Supplemental material for Effect of cat litters on feline coronavirus infection of cell culture and cats Click here for additional data file. Supplemental material, Cat_litter_Supplementary_Material_Apr_2019 for Effect of cat litters on feline coronavirus infection of cell culture and cats by Diane Addie, Lene Houe, Kirsty Maitland, Giuseppe Passantino and Nicola Decaro in Journal of Feline Medicine and Surgery
