# Supplementary material for: Effect of cat litters on feline coronavirus infection of cell culture and cats
Source: J Feline Med Surg. 2019 May 16;22(4):350–7. doi: 10.1177/1098612X19848167 (PMC8685581; doi:10.1177/1098612X19848167)
Supplement: Cat_litter_Supplementary_Material_Apr_2019 – Supplemental material for Effect of cat litters on feline coronavirus infection of cell culture and cats [file Cat_litter_Supplementary_Material_Apr_2019.docx]

# Fuller’s earth cat litters reduce feline coronavirus infection of cell culture and cats: Supplementary Material

D.D. Addie, Lene Houe, Kirsty Maitland, Giuseppe Passantino, Nicola Decaro

**Supplementary Table L**

**FCoV RNA RT-qPCR results for cats in Household L. during a cross-over controlled blinded trial.**

|  | **2009** | | | | | | **2010** | | | **2011** | | | | |  |  |  |  |  |  |
| --- | --- | --- | --- | --- | --- | --- | --- | --- | --- | --- | --- | --- | --- | --- | --- | --- | --- | --- | --- | --- |
|  | **Jan** | **Feb** | **Mar** | **Apr** | **Jul** | **Nov** | **Apr** | **Sep** | **Nov** | **Jan** | **Jun** | **17 Aug** | **31 Aug** | **Sep** | **Litter B** | | | **Litter X** | | |
| **Cat** | **Litter B** | | | | |  | **B** | **Litter X** | **Litter Y** | **Litter B** | | **Litter X** | | | **Pos** | **Total** | **%** | **Pos** | **Total** | **%** |
| **L1** | 3 | 2 | 0 | 3 | 0 | <1 | <1 | 2 | 2 | 2 | 2 | 0 | 0 | 2 | 7 | 9 | 78 | 2 | 4 | 50 |
| **L2** | 0 | 2 | 0 | 0 | 0 | 1 | 0 | 0 | 1 | 1 | 1 | 0 | <1 | 0 | 4 | 9 | 44 | 1 | 4 | 25 |
| **L3** | 2 | 2 | 0 | 2 | 0 | 1 | <1 | 1 | 2 | 0 | <1 | 0 | <1 | 0 | 6 | 9 | 67 | 2 | 4 | 50 |
| **L4** | 3 | 1 | 0 | 0 | 0 | 2 | 1 | 0 | 1 | 0 | 1 | 0 | 3 | 0 | 5 | 9 | 56 | 1 | 4 | 25 |
| **L5** | 0 | 0 | 0 | 0 | 0 | 0 | 0 | 0 | <1 | 0 | 1 | 0 | <1 | 1 | 1 | 9 | 11 | 2 | 4 | 50 |
| **L6** | 2 | 1 | 0 | 0 | 0 | 0 | <1 | 0 | 0 | <1 | 0 | 0 | 0 | 0 | 4 | 9 | 44 | 0 | 4 | 0 |
| **L7** | 2 | 0 | 2 | 0 | 2 | 0 | 0 | 0 | 1 | 0 | 1 | 0 | 0 | 2 | 4 | 9 | 44 | 1 | 4 | 25 |
| **L8** |  |  |  |  | New kitten | | <1 | 0 | 0 | 0 | 1 | <1 | 1 | 2 | 2 | 3 | 67 | 3 | 4 | 75 |
| **L9** | 1 | 2 | 2 | 0 | 1 | 0 | 0 | Euthanased | |  |  |  |  |  |  |  |  |  |  |  |
| **L10** |  |  |  |  |  |  |  | New kitten | | | 3 | 3 | 1 | 0 |  |  |  |  |  |  |

Cat names were substituted with numbers to preserve client confidentiality.

This table shows when a rectal swab was negative (0) or positive, with the log_10_ of feline coronavirus given, extrapolated from the FCoV RT-qPCR result. <1 indicates where log^10^ was zero, but a small amount of FCoV RNA, corresponding to up to 9 virus particles, was detected. Boxes containing positive results after a negative result, likely indicative of new infection or re-infection (i.e. virus transmission), are shaded. Virus transmission events were not significantly different between litter X and litter B (p = 0.2).

Pos = the number of positive rectal swabs.

Total = the total number of rectal swabs tested for each individual cat on each of the two cat litters.

% = the percentage of FCoV RNA positive swabs out of the total number taken while the cat was on each cat litter.

Cat L9 data was not included in the cross-over study because she was never on Litter X.

Kitten L10 was not included in the cross-over study analysis because of too few data points.

**Supplementary Table H**

**FCoV RNA RT-qPCR results for cats in Household H. during a cross-over controlled blinded trial.**

|  | **2008** | **2009** | | | | | | **2010** | | | **2011** | | | |  |  |  |  | |  |
| --- | --- | --- | --- | --- | --- | --- | --- | --- | --- | --- | --- | --- | --- | --- | --- | --- | --- | --- | --- | --- |
| **Cat** | **Sep** | **Jan** | **Feb** | **Mar** | **Apr** | **Jul** | **Nov** | **Apr** | **Sep** | **Oct** | **Jan** | **Jun** | **31 Aug** | **Sep** | **Pos** | **Total** | **%** | **Pos** | **Total** | **%** |
|  | **Litter A** | | | | | | | | **X** | **Y** | **Litter A** | | **Litter X** | | **Litter A** | | | **Litter X** | | |
| H1 | 2 | 2 | 0 | 3 | 2 | 1 | 0 | <1 | 0 | 1 | 3 | 1 | 0 |  | 7 | 9 | 78 | 0 | 2 | 0 |
| H2 |  | 2 | 4 | 0 | 2 | 1 | 1 | <1 | 2 | 0 | 1 | 2 | 1 | 0 | 8 | 9 | 89 | 2 | 3 | 67 |
| H3 |  | 0 | 4 | 0 | 0 | 0 | 1 | 1 | 0 | 2 | 1 | 1 | 0 | 0 | 5 | 9 | 56 | 0 | 3 | 0 |
| H4 |  |  |  |  |  | 0 | 0 | 0 | 0 | 1 | 1 | 1 | 0 | 0 | 2 | 5 | 40 | 0 | 3 | 0 |
| H5 | 0 | 0 - 3 | 3 | 0 | 0 | 0 | <1 | 0 | 0 | 1 | 0 |  |  |  | 3 | 9 | 33 | 0 | 1 | 0 |
| H6 | 0 | 2 | 0 | 2 | 0 | 0 | <1 | 0 | <1 | <1 | 2 |  |  |  | 4 | 8 | 50 | 1 | 1 | 100 |
| H7 | 0 | 0 | 3 | 0 | 2 | 0 | 0 | 0 | 0 | 0 | 2 |  |  |  | 3 | 8 | 38 | 0 | 1 | 0 |
| H8 | 1 | 3 | 0 | 0 | 0 | 0 | 0 | 0 | ND | 1 | 0 |  |  |  | 1 | 8 | 13 |  |  |  |
| H9 |  | 3 | 2 | 2 | 2 | 0 | 0 | 0 | 0 | 2 | 1 |  |  |  | 5 | 8 | 63 | 0 | 1 | 0 |
| H10 |  | 2 | 0 | 0 | 0 | 0 | 0 | 0 | <1 | 0 | 0 |  |  |  | 1 | 8 | 13 | 1 | 1 | 100 |
| H11 |  | 4 | 4 | 2 | 2 | 0 | **0** | 1 | 0 | 0 | 0 |  |  |  | 5 | 8 | 63 | 0 | 1 | 0 |
| H12 | 0 | 3 | 3 | 2 | 3 | 0 | **2** | 0 | 0 | 2 | Euthanased | |  |  | 5 | 7 | 71 | 0 | 1 | 0 |
| H13 |  | 3 | <1 | 3 | 2 | 0 | 0 | 1 | <1 | 2 |  |  |  |  | 5 | 7 | 71 | 1 | 1 | 100 |
| H14 | 0 | 2 | 2 | 3 | 2 | 2 | **0** | FIP |  |  |  |  |  |  |  |  |  |  |  |  |
| H15 | 0 | 0 | 2 | 0 | 1 | 0 | <1 | Euthanased | |  |  |  |  |  |  |  |  |  |  |  |
| H16 | 1 | 0 | 2 | 2 | 1 |  |  |  |  |  |  |  |  |  |  |  |  |  |  |  |
| H17 | 0 | 0 | 0 | 0 | 0 | 0 |  |  |  |  |  |  |  |  |  |  |  |  |  |  |
| H18 | 2 | 2 | 3 | 3 | 2 | 0 | **2** | Euthanased | |  |  |  |  |  |  |  |  |  |  |  |
| H19 | 1 | 0 | 2 | 3 | 3 | 2 | **0** |  |  |  |  |  |  |  |  |  |  |  |  |  |
| H20 | 0 | 1 | 0 | 0 | 3 | 0 | 0 | Euthanased | |  |  |  |  |  |  |  |  |  |  |  |
| H21 |  | 4 | 6 | 3 | 2 | 0 | 0 | Euthanased | |  |  |  |  |  |  |  |  |  |  |  |
| H22 |  | 2 | 3 | 0 | 3 | 1 |  |  |  |  |  |  |  |  |  |  |  |  |  |  |
| H23 |  | 2 | 2 | 3 | 2 | 0 | 0 |  |  |  |  |  |  |  |  |  |  |  |  |  |
| H24 | 1 | 2 | 2 | 3 |  |  |  |  |  |  |  |  |  |  |  |  |  |  |  |  |
| H25 |  | 3 | 4 | 3 |  |  |  |  |  |  |  |  |  |  |  |  |  |  |  |  |
| H26 |  | 4 | ND | 4 |  |  |  |  |  |  |  |  |  |  |  |  |  |  |  |  |
| H27 | 0 | 3 | 0 |  |  |  |  |  |  |  |  |  |  |  |  |  |  |  |  |  |
| H28 |  |  |  | New | 1 | 1 - 2 | **0** | 1 |  |  |  |  |  |  |  |  |  |  |  |  |
| H29 |  |  |  |  |  |  |  | 0 | <1 | 1 | Rehomed |  |  |  |  |  |  |  |  |  |
| H30 |  |  |  |  |  |  |  |  |  |  |  | 3 | 1 | 1 | 1 | 1 |  | 2 | 2 |  |
| H31 |  |  |  |  |  |  |  |  |  |  |  | 3 | 0 | 0 | 1 | 1 |  | 0 | 2 |  |
| H32 |  |  |  |  |  |  |  |  |  |  |  | 0 | 0 | <1 | 0 | 1 |  | 1 | 2 |  |
| H33 |  |  |  |  |  |  |  |  |  |  |  | 4 | 0 | 2 | 1 | 1 |  | 1 | 2 |  |
| H34 |  |  |  |  |  |  |  |  |  |  |  | 0 | 0 | 2 | 0 | 1 |  | 1 | 2 |  |
| H35 |  |  |  |  |  |  |  |  |  |  |  | 0 | 0 | 0 | 0 | 1 |  | 0 | 2 |  |
| H36 |  |  |  |  |  |  |  |  |  |  |  | <1 | 0 | 0 | 1 | 1 |  | 0 | 2 |  |
| H37 |  |  |  |  |  |  |  |  |  |  |  | 1 | 0 | 0 | 1 | 1 |  | 0 | 2 |  |
| H38 |  |  |  |  |  |  |  |  |  |  |  | 0 | 0 | 0 | 0 | 1 |  | 0 | 2 |  |
| H39 |  |  |  |  |  |  |  |  |  |  |  | 2 | <1 | 2 | 1 | 1 |  | 2 | 2 |  |
| H40 |  |  |  |  |  |  |  |  |  |  |  | 0 | ND | 0 | 0 | 1 |  | 0 | 1 |  |
| H41 |  |  |  |  |  |  |  |  |  |  |  | 2 | 0 | 0 | 1 | 1 |  | 0 | 2 |  |

ND = not done

Cat names were substituted with numbers to preserve client confidentiality.

September 2008 samples could not be used to compare Cat Litters A and X, because Litter A wasn’t begun until December 2008.

This table shows when a rectal swab was negative (0) or positive, with the log_10_ of feline coronavirus given, extrapolated from the FCoV RT-qPCR result. <1 indicates where log^10^ was zero, but a small amount of FCoV RNA, corresponding to up to 9 virus particles, was detected. Boxes containing positive results after a negative result, likely indicative of new infection or re-infection, are shaded. Cat H17 was identified as a possible FCoV resistant cat, so her results were not included in analyses. A greater proportion of negative cats remained virus free on litter X than on litter A (p = 0.02).

Pos = the number of positive rectal swabs.

Total = the total number of rectal swabs tested for each individual cat on each of the two cat litters.

% = the percentage of FCoV RNA positive swabs out of the total number taken while the cat was on each cat litter.
